# *Mycobacterium tuberculosis*-specific plasmablast levels are differentially modulated in tuberculosis infection and disease

**DOI:** 10.1016/j.tube.2020.101978

**Published:** 2020-09

**Authors:** Awa Gindeh, Olumuyiwa Owolabi, Simon Donkor, Jayne S. Sutherland

**Affiliations:** Vaccines and Immunity Theme, Medical Research Council Unit The Gambia at the London School of Hygiene and Tropical Medicine, Banjul, The Gambia

**Keywords:** Plasmablasts, ESAT-6/CFP10, Mtb infection spectrum, Immunology

## Abstract

**Background:**

While T cell responses to *Mycobacterium tuberculosis* (Mtb) have been extensively studied, the role of B-cells and antibodies are less well characterised. The aim of this study was to assess levels of Mtb-specific IgG + plasmablasts across the Mtb infection spectrum.

**Methods:**

Patients with active TB were analysed at baseline and 6 months of therapy (n = 20).Their exposed household contacts (HHC) included individuals with latent TB infection (LTBI; n = 20); evident at baseline; individuals with a negative Tuberculin Skin Test (TST) at baseline who became; positive at 6 months (converters; n = 11) and those who remained negative (non-converters; n = 10). An e x-vivo B-cell ELISPOT was performed to analyse plasmablast responses.

**Results:**

Frequencies of ESAT-6/CFP-10 (EC)- but not Whole Cell Lysate (WCL)-specific plasmablasts were significantly higher in patients with active TB pre-treatment compared to post-treatment (p = 0.002) and compared to HHC with LTBI (p < 0.0001). Conversely, total IgG + plasmablasts were significantly decreased in TB patients at baseline. No difference was seen in levels of plasmablasts between TST converters and non-converters at baseline.

**Conclusions:**

We show that EC-specific plasmablast levels are differentially modulated during TB infection and disease, with levels highest during active TB. These data provide new insights into TB biomarker development and avenues for novel immune interventions.

## 1 Introduction

Despite the availability of a vaccine and an effective anti-TB therapy, TB still remains a major global health problem [1]. The predominant contributing factors to the sustained epidemic are lack of a protective vaccine, HIV infection, diagnostic difficulties and emergence of multi and extensive drug resistant strains of *Mycobacterium tuberculosis* (Mtb) [2]. This resulted in 10 million newly diagnosed cases and 1.5 million deaths in HIV negative individuals in 2018 [3]. Interestingly, not all individuals exposed to Mtb develop clinical disease. About 90% of people will mount a protective immune response that contains the infection but rarely eliminates it – termed latent TB infection (LTBI) [4]. Approximately one-fourth of the global population are latently infected with TB [5], with 5–10% developing active disease in their lifetime [3].

Identification of immune subsets protective during different stages of Mtb infection and disease is imperative for development of more protective vaccines [6,7]. Some individuals exposed to Mtb may clear the infection through innate immune mechanisms but most require the adaptive immune system to contain the infection in granulomas [8]. This is predominantly orchestrated by T-cell subsets, mainly T helper (Th) 1 [8]. B-cells are also activated leading to the production of Mtb-specific antibodies; however, the role of B-cells and antibodies in TB immunity is yet to be fully elucidated [9].

Since we do not yet know the exact requirements for protective immunity to Mtb, it is crucial that previously underappreciated cells are evaluated [10]. The change in the frequency of the circulating B-lymphocyte repertoire during active TB disease remains controversial: some studies have reported either a significant decrease [[10], [11], [12]] or increase [13] of B-cells in the blood of patients with active TB. Data from South Africa showed a decrease in proportion of mature B cells from TB cases compared to other-lung diseases at diagnosis [10]. A subpopulation of activated memory B cells (CD19 + IgM + CD23 + CD27^+^) cells were found to be present at the end of TB treatment. A study from East Africa showed that Mtb-specific memory B-cells (MBCs) to BCG were significantly higher in vaccinated compared to unvaccinated individuals in Uganda [14]. The same group also showed elevated levels of Mtb-specific plasmablasts (IgG+) in active TB patients despite a reduction in MBCs compared to healthy and LTBI individuals [15]. Another study from Europe showed that patients with active TB had reduced circulating B-cell frequencies with impaired proliferation, immunoglobulin- and cytokine-production. These defects disappeared upon successful treatment [16]. Plasmablasts are short-lived plasma cells mainly present in the peripheral circulation following an active or ongoing infection. A study from Ethiopia demonstrated that circulating IgG + plasmablasts and spontaneous secretion of BCG-specific IgG antibodies were significantly higher in patients with active TB compared with latent TB cases and non-TB controls [17]. Their study concluded that the ratio of plasmablasts and MBCs could be used as a potential biomarker to predict clinical status of TB infected individual living in TB endemic settings [17].

Due to the genetic differences in both host and pathogen in West Africa, it is unknown how these findings translate. Thus, the aim of this pilot study was to determine the frequency of Mtb-specific and non-specific IgG plasmablasts in Mtb infection and disease in a West African setting.

## 2 Methods

### 2.1 Ethics statement

Ethical approval was obtained from the Medical Research Council/Gambia government joint ethics committee (SCC1333). All study participants provided written informed consent for the collection of samples and subsequent analysis.

### 2.2 Study participants

Twenty (20) GeneXpert and culture positive first episode TB patients were analysed pre and post TB treatment (baseline and 6 months). In addition, 20 tuberculin skin test (TST) positive (defined as LTBI) asymptomatic (assessed by chest x-ray and symptom screening) household contacts, 11 TST converters (ie TST negative at baseline but TST positive at follow-up) and 10 non-converters (TST negative at both time-points) were analysed at baseline only. All study participants were HIV negative adults.

### 2.3 Antigens

ESAT-6/CFP-10 (EC) fusion proteins were kindly provided by Prof. Tom Ottenhoff (Leiden University Medical Center). Whole cell lysate (WCL) was obtained from BEI (USA). All antigens were used at a final concentration of 10 μg/ml.

### 2.4 PBMC isolation, storage and thawing

Peripheral blood mononuclear cells (PBMCs) were isolated using density gradient centrifugation and stored in liquid nitrogen until needed. The PBMCs were then removed from liquid nitrogen and semi-thawed in a 37 °C water bath. The cells were transferred into a 15 ml falcon tube containing pre-warmed RPMI (Sigma, UK) +10%FCS (Sigma, UK) +200U/ml streptomycin+2 mM l-glutamine (R10) and centrifuged at 1500 rpm for 7 min. Following centrifugation, the supernatant was decanted and the cells resuspended in 5 mls R10 + 0.02% Benzonase (Sigma, UK) and rested for 5–6 h at 37 °C, 5% CO_2_. After this incubation, the cells were washed and resuspended in 2 mls R10+Benzonase for counting.

### 2.5 *Ex-vivo* ELISPOT assay

An *ex-vivo* B-cell ELISPOT assay was used to determine the frequencies of *Mtb*-specific plasmablasts. Ninety-six well filter ELISPOT plates (Millipore, Germany) were coated overnight with 100 μl of PBS containing Mtb-specific proteins. Fragment donkey anti-human IgG (10 μg/ml) was used as a positive control while wells coated with PBS only served as negative controls. Plates were washed and blocked with R10 for 2 h 200,000 cells were added into the antigen-coated and negative control wells in triplicate. For the anti-human IgG coated wells, a 1:200 dilution of the cell suspension was made and 50 μl was added to the ELISPOT plates already containing 150 μl R10 (4000 cells/well final). The plates were then incubated at 37 °C with 5% CO_2_ overnight. Plates were washed with PBS and incubated with biotin-streptavidin peroxidase-conjugate (Jackson ImmunoResearch, USA) for 2 h. The plates were washed again and 100 μl streptavidin- Alkaline-phosphatase (1:1000) was added to the plates and incubated for 1 h. Alkaline-phosphatase (AP)-conjugate was then added for 10 min in the dark. Developed plates were air-dried and the numbers of resultant spots were counted on an Autoimmun Diagnostika (AID) ELISPOT plate reader (version 7.0). Frequencies of antigen-specific plasmablasts were calculated automatically and data presented as spot forming units (sfu) per million PBMCs.

### 2.6 Statistical analysis

Graph Pad Prism version 7.0 (Software MacKiev, USA) was used for the statistical analysis. Prior to analysis, background (Nil) responses were subtracted from the stimulated wells. Comparisons of antigen-specific PB responses between active TB cases pre-treatment (recruitment) and post-treatment (6-months) and TST + contacts were made using the Kruskal-Wallis test followed by Dunn's post-test comparison. Analysis of TB cases pre- and post-treatment was performed using the Wilcoxon-matched pairs test. A two tailed T-test was used to analyse responses between the converters and non-converters. A p value ≤ 0.05 was considered significant.

## 3 Results

### 3.1 Participant demographics

Participant samples were selected to ensure even distribution of age and sex between the groups (Table 1). The median [IQR] range for participants with active TB (ATB), latent TB (LTBI), TST converters and non-converters was 27[21–42], 28[21–43], 30[23–60] and 22[18–38] respectively. The % male in each group was 45, 35, 53 and 50 respectively. All participants were HIV negative.

**Table 1:** Participant demographics.

	ATB	LTBI	TST Converters	TST Non-converters
N =	20	20	11	10
Age	27[21–42]	28[21–43]	30[23–60]	22[18–38]
Males (%)	45	35	53	50

N = number; Age (median[interquartile range]); ATB, active TB; LTBI, latent TB infection; TST, tuberculin.

Skin test; TST converters, TST negative at baseline and positive at 6 months; TST non-converters, TST negative at baseline and 6 months.

### 3.2 Plasmablast levels in active TB disease compared to LTBI

**Fig. 1:**
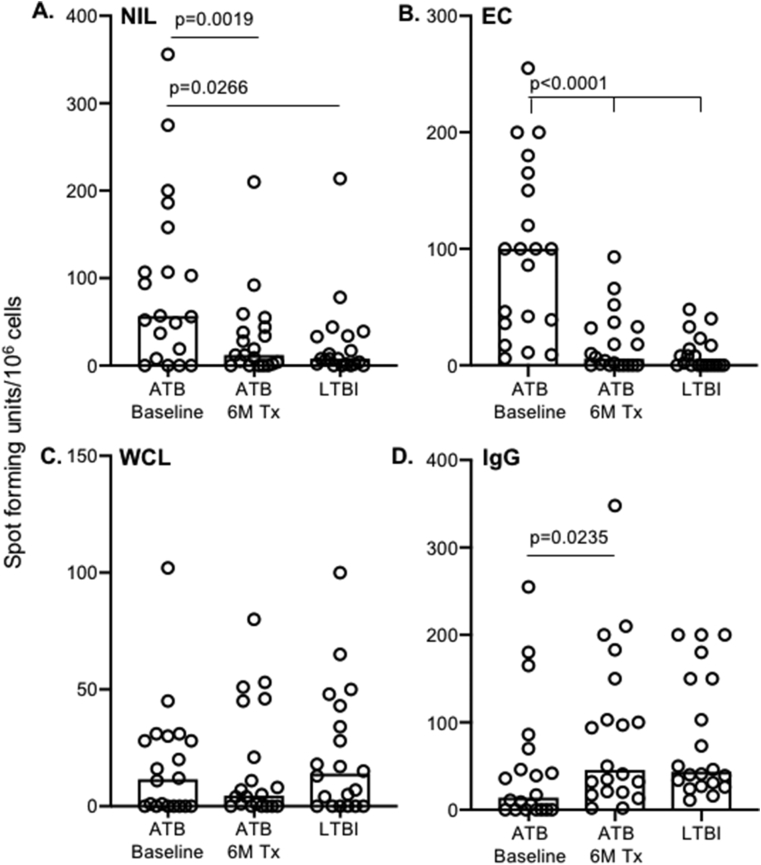
**Plasmablast responses in active TB cases before and after therapy and in LTBI**. Plasmablast responses were analysed following short-term *ex-vivo* ELISPOT. **A**: background (NIL); **B**: ESAT-6/CFP-10 (EC); **C**: Whole Cell Lysate (WCL) and **D**: Total IgG+ producing cells. Data analysed by Kruskal-Wallis with Dunn's post-test comparison. Box height indicates median.

In the absence of stimulation (NIL), plasmablast levels were significantly higher in TB cases at recruitment compared to 6 months of treatment and to LTBI levels (p = 0.0019 and p = 0.0266 respectively; Fig. 1). No difference was seen between levels at 6 months of treatment and LTBI (Fig. 1). Following EC stimulation (with background subtraction), plasmablast levels were significantly higher in TB cases at recruitment compared to post-treatment and LTBI (p < 0.0001 for both; Fig. 1). Again, no statistical difference in EC PB responses was observed between TB cases after 6-months of therapy and individuals with LTBI. There was no difference in responses to WCL nor total IgG between TB cases and LTBI at baseline nor for WCL between TB cases at baseline and 6 months (Fig. 1). However, responses to total IgG were significantly higher after 6 months of therapy compared to active TB at baseline (p = 0.0235; Fig. 1). When the ratio of EC-specific to total IgG responses were analysed, TB cases at recruitment had a significantly higher ratio compared to both cases at 6 months of tx and LTBI (p < 0.0001 for both; data not shown).

### 3.3 Plasmablast responses in TST converters and non-converters

**Fig. 2:**
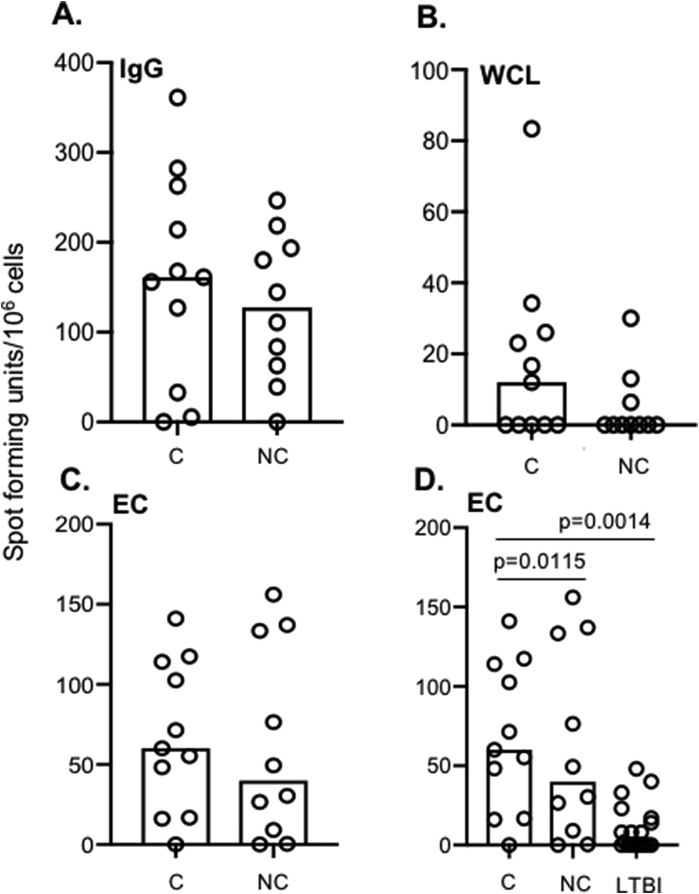
**Plasmablast responses in TST converters (C) and non-converters (NC)**. Antigen specific PBs were determined by short-term *ex-vivo* ELISPOT. **A**: Total IgG; **B**: Whole cell lysate (WCL); **C**: ESAT-6/CFP-10 (EC) and **D**: EC stimulation in NC and C compared to LTBI. A-C analysed using Mann-Whitney U-test. D analysed using Kruskal-Wallis with Dunn's post-test comparison. Box height indicates median.

TST converters and non-converters were analysed at the baseline time-point (prior to conversion). While the median responses to all antigens except EC were higher in the converters, none reached statistical significance (Fig. 2A–C). Interestingly, no difference in levels of IgG or WCL responses were seen between C, NC and LTBI at baseline but a significantly higher level of EC-specific plasmablasts were seen in both C and NC at baseline compared to LTBI (p = 0.0115 and p = 0.0014 respectively; Fig. 2D).

## 4 Discussion

In this study we have shown that peripheral Mtb*-*specific plasmablast responses are differentially modulated at key control points within the Mtb infection spectrum. EC-specific PB responses were initially high in early infection, reduced in latency, significantly increased during active TB disease and reduced following successful TB treatment to LTBI levels. The high levels of plasmablasts in patients with active TB, despite an overall reduction in total IgG plasmablast levels, is likely due to an increase in mycobacterial loads resulting in preferential expansion of Mtb-specific plasmablasts despite a reduction in the total pool available [15]. This may also activate Mtb-specific memory B-cells (MBCs), which subsequently differentiate into new plasmablast populations, thereby contributing to the increased pool of plasmablasts and EC-specific IgG antibodies detected within the periphery [18,19]. Our results support previous findings that patients with active TB have a dysfunctional circulating B-cell population, including impaired proliferation, Ig and cytokine production, which normalises following TB treatment [20,21]. Indeed, we saw a significant decrease in total IgG plasmablasts during active TB despite an increase in Mtb-specific levels, which was resolved with treatment.

We have previously shown a predominant B cell signature in individuals who remained TST negative compared to those who were TST negative at baseline but converted to positive at a later time-point [22]. This suggested that B cells may play a role in early protection to Mtb infection and prompted us to investigate plasmablast levels in these same individuals. We found no difference in total IgG or EC-specific plasmablast levels at baseline in those who remained TST negative (non-converters) compared to those who converted. In contrast to findings from Uganda [15] however, levels of EC-specific plasmablasts were found to be higher in individuals who were TST negative at baseline compared to those who were TST positive (latently infected). These results suggest that analysis of other B cell subsets together with functionality would be of importance to gauge if B cells and/or antibodies provide a level of protection early in infection. For example, differential antibody glycosylation has been shown to occur preferentially on the Fc domain, providing significant discriminatory power between different states of *M. tuberculosis* infection and disease [23].

Our results indicate that Mtb-specific plasmablasts may serve as a valuable and early diagnostic marker for active TB disease. A memory B-cell ELISPOT should also be performed to determine if the plasmablast:MBC ratio can distinguish between active and latent TB [15] since PBs are significantly higher in active disease while MBCs are higher in latent TB infection. Thus, the ratio is more representative of the specific B-cell immune response to Mtb since MBCs also contribute to the plasma cell pool [15].

Taken together, these data demonstrate that peripheral blood B-cell responses are differentially modulated during active and latent TB infection, and that the frequencies of Mtb-specific PBs in peripheral blood may be able to accurately predict the clinical status of Mtb infected individuals living in highly TB-endemic regions. These data provide new insights into TB biomarker development and avenues for novel immune interventions.
